# Randomized Controlled Trials of HIV/AIDS Prevention and Treatment in Africa: Results from the Cochrane HIV/AIDS Specialized Register

**DOI:** 10.1371/journal.pone.0028759

**Published:** 2011-12-15

**Authors:** Babalwa Zani, Elizabeth D. Pienaar, Joy Oliver, Nandi Siegfried

**Affiliations:** 1 South African Cochrane Centre, Medical Research Council of South Africa, Cape Town, South Africa; 2 Department of Public Health and Primary Care, University of Cape Town, Cape Town, South Africa; Tulane University, United States of America

## Abstract

**Introduction:**

To effectively address HIV/AIDS in Africa, evidence on preventing new infections and providing effective treatment is needed. Ideally, decisions on which interventions are effective should be based on evidence from randomized controlled trials (RCTs). Our previous research described African RCTs of HIV/AIDS reported between 1987 and 2003. This study updates that analysis with RCTs published between 2004 and 2008.

**Objectives:**

To describe RCTs of HIV/AIDS conducted in Africa and reported between 2004 and 2008.

**Methods:**

We searched the Cochrane HIV/AIDS Specialized Register in September 2009. Two researchers independently evaluated studies for inclusion and extracted data using standardized forms. Details included location of trials, interventions, methodological quality, location of principal investigators and funders.

**Results:**

Our search identified 834 RCTs, with 68 conducted in Africa. Forty-three assessed prevention-interventions and 25 treatment-interventions. Fifteen of the 43 prevention RCTs focused on preventing mother-to-child HIV transmission. Thirteen of the 25 treatment trials focused on opportunistic infections. Trials were conducted in 16 countries with most in South Africa (20), Zambia (12) and Zimbabwe (9). The median sample size was 628 (range 33-9645). Methods used for the generation of the allocation sequence and allocation concealment were adequate in 38 and 32 trials, respectively, and 58 reports included a CONSORT recommended flow diagram. Twenty-nine principal investigators resided in the United States of America (USA) and 18 were from African countries. Trials were co-funded by different agencies with most of the funding obtained from USA governmental and non-governmental agencies. Nineteen pharmaceutical companies provided partial funding to 15 RCTs and African agencies co-funded 17 RCTs. Ethical approval was reported in 65 trials and informed consent in 61 trials.

**Conclusion:**

Prevention trials dominate the trial landscape in Africa. Of note, few principal investigators and funders are from Africa. These findings mirror our previous work and continue to indicate a need for strengthening trial research capacity in Africa.

## Introduction

Combating HIV/AIDS relies on the prevention of new infections and on providing effective antiretroviral therapy to patients with disease. In 2008, there were an estimated 33.4 million people living with HIV, 2.7 million new infections and 2 million HIV/AIDS related deaths. Only 33% of HIV-infected women received antiretroviral drugs to reduce the risk of mother-to-child transmission of HIV. Africa is the region most affected by HIV/AIDS [1]. It is thus critical that research is carefully conducted and responds to the health priorities of the continent. This is particularly important in Africa as the economic resources of the continent are limited [2].

The randomized controlled trial (RCT) is the gold standard for evaluating effects of healthcare interventions [3]. Researchers, health workers, policy-makers and consumers need information on planned, ongoing and completed clinical trials to enable them to effectively assess interventions for preventing or treating HIV/AIDS and related conditions and to plan future research. Our previous work [4] provided a descriptive analysis of RCTs of HIV/AIDS interventions conducted in Africa and reported up to the end of 2003. The current study updates that analysis with RCTs published from the beginning of 2004 up to the end of 2008. This new data will inform African stakeholders of gaps in research and highlight current achievements.

### Objectives

To identify, describe and analyze RCTs of HIV/AIDS interventions conducted in Africa and reported between 2004 and 2008.

## Methods

### Maintenance of the Cochrane HIV/AIDS Specialized Register

The Register comprises trial records stored in an MS Access database and is maintained by a dedicated information specialist and an assistant. We conduct quarterly searches of two major electronic databases, PUBMED and EMBASE, using the Cochrane highly sensitive search strategy (Appendix S1) for retrieving RCTs [5] coupled with a comprehensive HIV/AIDS search string (Appendix S2) [4]. We search the Cochrane Central Register of Controlled Trials (CENTRAL) once a year. Four independent hand-searchers with epidemiological training identify any HIV/AIDS RCTs and controlled clinical trials in the search results for inclusion in the Register. A senior epidemiologist conducts quality control on a random 10% sample of these records. In September 2009, we used the built-in search tool of the Register to identify records coded as RCT and published between 2004 and 2008.

### Searching for trials, data extraction and analysis

We searched the Cochrane HIV/AIDS Specialized Register of trials (the Register). The abstracts retrieved were exported from the Register into ProCite and printed. Each abstract was reviewed by two independent researchers to identify RCTs conducted in Africa between 2004 and 2008. Full-text articles were obtained for potentially eligible RCTs and those for which we were uncertain. Two researchers independently read the articles and determined final inclusion according to the criteria in Table 1. Eligibility for studies that were unclear was verified by the third researcher. Data were independently extracted and compared by two researchers using a standardized data-extraction form and discrepancies were resolved with the third researcher. The third researcher also conducted quality control on a 10% sample selected through a random number generator in MS Excel.

**Table 1:** Eligibility criteria for Randomized Controlled Trials included.

	*Inclusion criteria*	*Exclusion criteria*
Intervention	Efficacy or effectiveness of HIV/AIDS specific interventions including pilot studies	Safety and acceptability trials
	Efficacy or effectiveness of non-HIV/AIDS specific interventions, but in or with a subgroup (at least 10%) of HIV infected participants	Trials measuring behavioral interventions without HIV incidence
Location	Conducted wholly or partly in Africa	Trials conducted in Africans living outside the continent
Participants	Infected with HIV-1, HIV-2 or dually infected, or in case of prevention trials, HIV uninfected but at risk of infection.	
Trial date	Reported between 2004 and 2008, if preliminary data only, authors will be contacted for additional results. Data on ongoing trials will not be extracted until their completion.	

Extracted data included details of principal investigators, trial location, details of interventions and methodological quality of RCTs (Table 2). Data were single-captured in MS Access and descriptively analyzed.

**Table 2:** Data extraction items in included trials.

Item	Details recorded
Reference	Trial ID; trial title; publication details
InterventionDates	Prevention; treatmentTrial start and end dates; duration of follow-up; early termination
Location	Single or multi-centre; city; region; country; details of other countries if multinational.
Principal investigator	Name; affiliation; qualifications; country of residence; address where available
Funders	Location (African or non-African); government agency; non-governmental agency; pharmaceutical company
Ethical approval	Location (African or non-African); method of informed consent
Methods	Sample size; power calculation; generation of allocation sequence; allocation concealment; blinding; loss to follow-up

## Results

**Figure 1:**
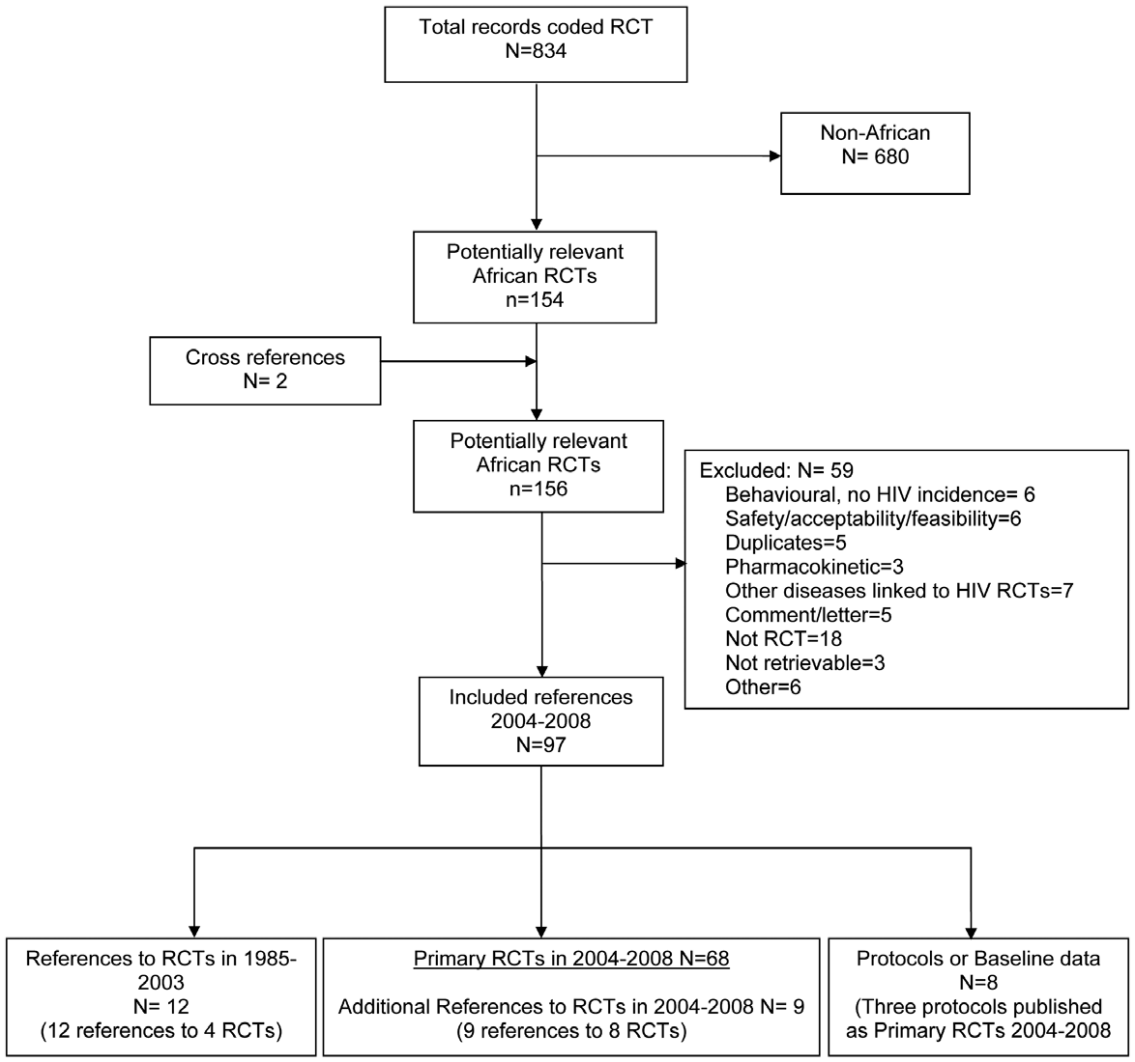
Flow diagram showing the assessment of African trials for 2004–2008 in the HIV register.

Our search for “RCTs” in the Register identified 834 records. Of these, 154 references described potentially relevant African RCTs. The remaining 680 references referred to RCTs conducted in non-African countries. With the inclusion of two additional cross references, the number of potential African RCTs increased to 156. We were unable to trace the reference for one study [6], and could not obtain full reports for two [7], [8], thus these three studies were excluded. After assessing eligibility, 97 references were included. Figure 1 shows the flow diagram including the reasons for exclusion of 59 references.

Of the 97 references included, 68 were primary RCTs reported for the first time during 2004–2008. Eight of the 68 primary RCTs had nine more references published during the same period. Twelve references reported on four RCTs already published before 2004 and included in the previous analysis [4]. We identified eight references to published protocols, three of which had primary RCTs published in the period under study. We extracted data on the 68 primary RCTs. Where an RCT was reported in more than one reference, we used data contained in all the related references.

### Sources of Trials

All trials included in the analysis were published as journal articles. Nine were published in *The Lancet*, seven in *AIDS* and six in the *New England Journal of Medicine*. Other journals included the *American Journal of Clinical Nutrition* and *Journal of Infectious Diseases* with four articles each; the *American Journal of Obstetrics and Gynecology, BMJ, PLoS ONE* and the *Journal of Acquired Immune Deficiency Syndromes* had three articles each. The remaining 26 trials were published in 20 different journals. The 2008 impact factor for the journals with one published article ranged between 1.517 and 7.069 (seven journals). Five journals with two published articles each had an impact factor range between 2.304 and 31.718. The remaining eight journals were not listed in the Institute for Scientific Information Web of Knowledge Journal Citation Report for 2008. All study reports were published in English language journals. Sources of trials are shown in Appendix S3.

### Location and centre types of African RCTs

Seven RCTs were conducted at multiple sites across 11 countries which included Benin, Cameroon, Ethiopia, Ghana, Malawi, Nigeria, South Africa Tanzania, Uganda Zimbabwe and Zambia. Thirty-six RCTs were conducted in multiple centres in a single country. Of these, South Africa hosted 12 trials, Tanzania and Zambia hosted five trials each, and Malawi hosted four trials, Kenya, Nigeria and Zimbabwe hosted two trials each. Botswana, Burkina-Faso, Rwanda and Uganda each hosted one multi-site trial each.

**Figure 2:**
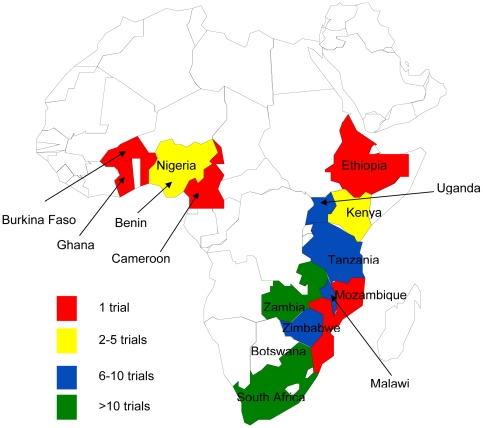
The countries where the trials were conducted.

Twenty-five trials were single-centre RCTs with five conducted in South Africa and five in Zambia. Four trials single-centre trials were conducted in Zimbabwe, three were conducted in Kenya and Uganda each and Malawi hosted two trials. Mozambique, Nigeria and Tanzania hosted one trial each. Figure 2 shows the map of African countries and the number of trials conducted per country.

### Principal investigators

The principal investigator (PI) was clearly reported in 21 of the 68 trials. In 17 of the trials the PI was also the corresponding first author and in one trial the corresponding last author. The PI was the first author in one and the last author in two of the remaining trials but not the corresponding author.

When the PI was not clearly stated, we classified the first author as the PI for further analysis. Most principal investigators, both the clearly stated and the assumed, were based in the USA (29) and in the United Kingdom (10), with 18 PIs residing in Africa: in South Africa (8), Zambia (5) Uganda (2), Kenya (1), Rwanda (1), and in Nigeria (1). The other non-African PIs resided in Denmark (3), France (1), Netherlands (1), Canada (1) and India (1). The locations of four PIs could not be identified.

Qualifications of the PIs were generally not reported. Of the 18 reported, seven had PhD degrees, six medical degrees, three had both medical and PhD degrees and two had medical and Masters degrees.

### Trial types

Forty-three RCTs assessed interventions for the prevention of HIV and related infections, while 25 assessed interventions for the treatment of AIDS and related infections.

#### Prevention trials

Of the 43 prevention trials, 15 (35%) investigated the prevention of mother-to-child transmission of HIV. Behavioural interventions and microbicides were investigated in five trials each. Six trials investigated the use of pharmaceutical products for the prevention of opportunistic infections in HIV-infected participants, while one trial evaluated pharmaceutical products for HIV. Other trials investigated the effects of male circumcision (3) and nutritional interventions in HIV-infected participants (4). Four other trials investigated interventions to improve the uptake of HIV counselling and testing, the use of contraception methods, planning for the future by HIV-infected couples and the prevention of malaria in HIV-infected patients. We did not identify any trials for vaccine efficacy.

#### Treatment trials

Twelve (48%) of the 25 treatment trials focused on pharmaceutical products for the treatment of opportunistic infections and three investigated pharmaceutical products for the treatment of AIDS. Others investigated the use of pharmaceutical products for other infections in HIV-infected people (3), the effects of nutritional interventions (4), the effects of exercise (1), delivery of highly-active antiretroviral therapy (HAART) by direct observation (1) and interventions for treating bacterial vaginosis (1).

### Trial Dates

Twenty-one trials reported both the month and year participant enrolment into the trial began and ended, and the month and year the trial was completed. Nineteen reported the month and year when participant enrolment began and ended, but did not report the trial completion dates. Twelve reported the month and year the trial started and ended, but did not report the dates of participant enrolment. Ten RCTs reported some dates, e.g. trial start and end years with no months or the month and year the trial started without reporting the end dates. In six of the trials no dates were reported.

The first trial commenced in 1994 and investigated the treatment of AIDS-associated Kaposi's sarcoma in Zimbabwe [9]. Nine trials commenced before the year 2000. The last five trials began in 2005 and two of these ended in 2007.

### Sample size, power calculation and primary outcome

**Figure 3:**
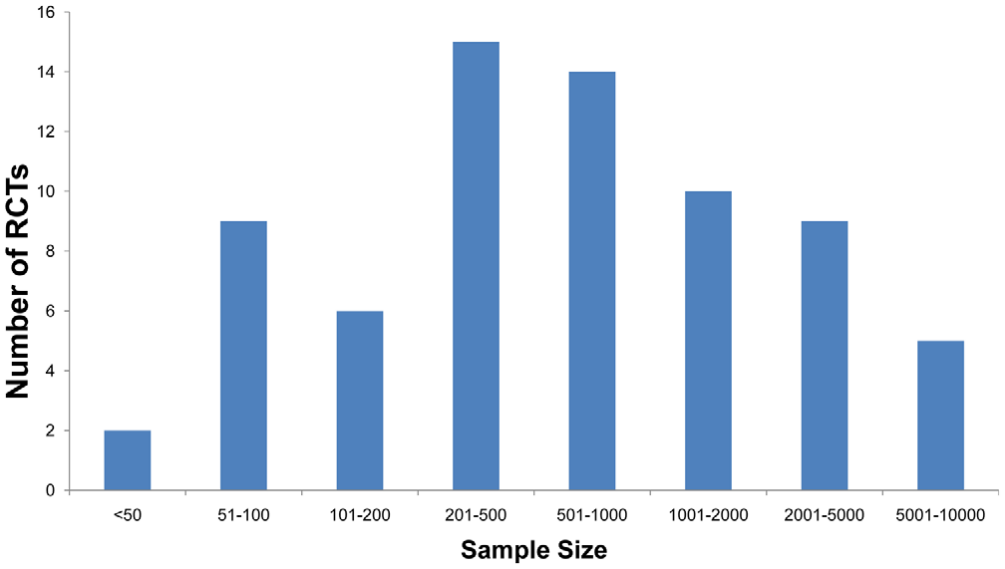
The ranges of the sample size in the trials.

The median sample size was 626 participants. The sample size ranged from 33 participants in a trial investigating the treatment of Kaposi's sarcoma conducted in Durban, South Africa [10] to 9645 participants in a behavioural intervention trial conducted in the Mwanza region in Tanzania [11]. In 31 RCTs, the number of participants was less than or equal to 500, with four RCTs including more than 5000 participants (see Figure 3).

In 54 trials, the sample size and power calculation based on the primary outcome was conducted before the trial began. In fourteen trials there was no report of power calculation. The primary outcome was clearly indicated in 56 trials, while 12 trials did not report which of the outcomes was the primary outcome.

### Number of intervention groups and types of randomization

Fifty-seven trials compared two interventions, six compared three and five compared four interventions with each other. In 43 trials participants were randomized at the individual level, while 17 trials randomized mother and child pairs. One trial randomized couples. There were six cluster trials and one cross-over trial. Three cluster trials investigated behavioural interventions, two investigated the prevention of other infections in HIV-infected participants and one investigated the use of pharmaceutical products for the prevention of opportunistic infections in HIV- infected participants.

### The quality of methods

#### Generation of the randomization sequence

We judged the methods used to generate the random sequence to be free of bias in 38 trials. The methods used to generate the randomization sequence included a computer-generated list (31), a randomized list generated by a statistician (2), a random number list/table (2), and a randomized list prepared off-site (1). In one trial, the methods were only described as permuted without any further description [12] and coin tossing was used in one trial [13]. In 30 trials we could not determine the methods used to generate the random sequence.

#### Allocation concealment

Methods used for allocation concealment was assessed to be free of bias in 35 trials. These included the use of sequentially numbered, sealed, opaque or padded envelopes in 14 trials, treatment given in identical, non-transparent and sequentially numbered containers (11), the randomization list kept in sealed envelopes drawn independently and sequentially (5), the code or the list held off-site with no access by the investigators (4) and centralized randomization (1). Methods for allocation concealment were not adequately described in 32 trials. The allocation sequence was inadequately concealed in one trial, where the first 17 participants were assigned to one intervention and randomization started on the 18^th^ participant.

#### Blinding of providers, participants and outcome assessors

Eighteen trials clearly stated that providers, participants and outcome assessors were blinded to the interventions into which the participants were allocated. In six trials, providers and participants were blinded, but it was unclear whether the outcome assessors were blinded. Twenty-four trials blinded the providers, 33 blinded the participants and 31 blinded the outcome assessors. Providers were not blinded in 30 trials and participants were not blinded in 29 trials, however, outcome assessors were blinded in 12 of these trials. Table 3 shows the blinding of providers, participants and outcome assessors.

**Table 3:** Blinding of providers, participants and outcome assessors.

Provider	Participant	Assessors	Trials
Yes	Yes	Yes	18
Yes	Yes	Unclear	6
Unclear	Yes	Yes	1
Unclear	Yes	Unclear	8
Unclear	Unclear	Unclear	4
Unclear	No	Yes	1
No	Unclear	Yes	1
No	Unclear	Unclear	1
No	No	Yes	10
No	No	Unclear	14
No	No	No	4

### Consort flow diagram

A CONSORT flow diagram was included in 58 trials, clearly showing loss to follow-up. In 10 trials, the CONSORT flow diagram was not included.

### Early termination

Thirteen trials were terminated earlier than planned. Reasons for early termination included a significant effect of the intervention detected during data monitoring [14]–[18] and no significant effect during data monitoring [19]–[21]. In two of these trials, the results of data monitoring were confirmed in a recently published trial employing the same intervention [20], [21]. A possible increase in the risk in the intervention arm was cited in two trials [22], [23], in both, the decision to review the data was taken due to the publication of a related trial. In one trial [24], the US Food and Drug Administration recommended against the prolonged use of Nevirapine by women with CD4 counts ≥250 cells/mm^3^, thus the intervention was terminated. Non-compliance with the protocol [25] and low event rate of the outcome of interest [26] were cited as reasons in one trial each.

### Ethics approval and informed consent

Sixty-six trials reported receiving ethical approval and two trials did not report on ethical approval. Fifty-seven received approval from both African and non-African ethics review committees, eight reported receiving ethical review from African countries without mentioning international ethics review-boards, while one trial reported receiving ethical approval but did not state the name and location of the approving body. Of the eight trials that reported ethical approval only from African bodies, seven reported non-African funding agencies. Written informed consent was obtained from participants in 52 trials, and one trial obtained consent orally. Eight trials reported receiving consent, but the type of consent was not specified. Informed consent was not reported in seven trials.

### Funding

Fifty-eight trials were funded by multiple organisations and the primary funder could not be identified. Nine trials had a single funder. One trial [27] did not report on funding.

#### Government Agencies

Eight RCTs were funded by African government agencies; five agencies were from South Africa (National Institute for Communicable Diseases; National Research Foundation; Medical Research Council of South Africa; University of Cape Town and Rhodes University), two from Zimbabwe (Ministry of Health and Child Welfare, and University of Zimbabwe) and one from Rwanda (Multi-sectoral AIDS Program). None of the trials was solely funded by African government agencies. The majority of funding was obtained from non-African governments. Table 4 shows the details of non-African government agencies funding the trials.

**Table 4:** Non-African Government agencies providing funding to trials.

Country	Government agency	Trials
**Canada**	Canadian Institute for Health Research	1
	Canadian International Development Agency	1
	St. Michael's Hospital	1
**Denmark**	Council for Developmental Research	1
	Danish International Development Agency	2
	Danish Council for Medical Research	1
	Danish Embassy in Zimbabwe	1
**France**	Institute Nationale de la Sante et de la Rocherche Medicale	1
**Ireland**	Development Cooperation Ireland	1
**Switzerland**	Swiss National Science Foundation	1
	World Health Organization	1
**Unclear**	Angece Nationale de Recherches sur le SIDA	1
	Commission Nationale le Lutle Centre le SIDA	1
	Hearst Fellowship	1
**United Kingdom**	Department for International Development	5
	European Commission	1
	UK Medical Research Council	5
**USA**	Case Western Reserve University	1
	Centre for Disease Control and Prevention	4
	University of Connecticut	1
	Department of Agriculture	1
	Department of Health and Human Services	1
	National Institute of Health	
	National Institute for Drug Research	1
	National Institute of Allergy and Infectious Diseases	8
	National Institute of Child Health and Human Development	8
	National Institute of Drug Abuse	2
	National Institute of Health (department not specified)	22
	National Institute of Mental Health	4
	Office of AIDS research (for various NIH branches and for Universities)	2
	US President's Emergency Plan For AIDS Relief	2
	New York State's Tuition Assistance Programme	1
	UN Children's fund	1
	Joint United Nations Programme of HIV/AIDS	2
	United States Agency for International Development	11

#### Non-governmental agencies

Five African non-governmental agencies funded four trials; from South Africa (Africa Centre for Health and Population Studies), Malawi (Blantyre Christian Centre, and Wellcome Trust Laboratories in Malawi) and Zimbabwe (Jewish Humanitarian & Relief Committee and The Salvation Army). Non-African non-governmental agencies included Fogarty International (8), Wellcome Trust (8), and the Bill and Melinda Gates Foundation (4). One trial [17] was exclusively funded by a non-governmental agency (Rockefeller Foundation). USA based non-governmental agencies provided funding to 33 trials, United Kingdom based agencies funded eight, while Denmark and Canada based agencies funded five and one, respectively. The locations of 18 non-governmental agencies were unclear.

#### Pharmaceutical and other commercial companies

None of the trials were exclusively funded by pharmaceutical companies. Fourteen pharmaceutical companies provided funding to 16 trials. Funding from pharmaceutical companies included the provision of drugs, placebo or both. Nine of the trials that received funding from pharmaceutical companies were co-funded by the US government. One trial [28] was exclusively funded by a commercial company, Nestle SA.

## Discussion

This study provides a descriptive analysis of African HIV/AIDS RCTs published from January 2004 to December 2008, and updates our previous work where we reviewed RCTs published up to December 2003. The trial reports were obtained from the Cochrane HIV/AIDS Specialised Register and RCTs conducted partially or wholly in Africa were included.

### Number of trials, investigators and funders

This study identified more trials from South Africa and Zambia followed by Zimbabwe, Tanzania and Uganda. This is similar to the findings of a previous study of all RCTs conducted in sub-Saharan Africa and published up to 1999, which identified that almost half of the trials were conducted in South Africa [29]. This most likely reflects the dominant economic role South Africa plays on the continent and is not only a reflection of prevalence. Although Swaziland and Lesotho have higher prevalence of HIV compared to other African countries (26.1 and >23%, respectively), no trials were identified from these countries [1]. The low number of trials in some African countries with high HIV prevalence raises concerns about the interests of African governments and the evidence that drives local policy. Other factors that may hinder investigators from conducting trials in resource-constrained countries are difficulties in obtaining culturally relevant and well understood informed consent, ethical approval by governing bodies and the availability of infrastructure [30]–[32].

African countries often host trials that are led by non-African researchers [33]. In our study, only 18 (28%) of the principal investigators (PIs) were based in the continent. Most PIs were based in the USA and these findings were similar to previous observations [4], [34]. This is likely to indicate a need for capacity development of local investigators in trials research with the ultimate aim to ensure the research agenda of the African continent is driven from within. Non-African based researchers often conduct research in Africa on behalf of external agencies in collaboration with African researchers. White argues that these collaborations can encourage the transfer of skills to African researchers and ensure that the interests of the host country are considered when a trial is conducted [33]. The transfer of skills could further be enforced by the collaboration of experienced investigators with new researchers. Of the eighteen principal investigators with reported qualifications, nine had PhD degrees. The management of research projects and research grants requires substantial skills and experiences [35], however, we were unable to assess whether the qualifications of PIs have any impact on the conduct of trials as the qualifications of PIs were mostly not reported.

In our study, a number of the trials were funded by more than one organization, with most funding obtained from USA governmental agencies followed by United Kingdom governmental agencies. Few trials were funded by African organizations, in particular African governments. This is similar to our previous study which also observed few funders from the continent [4]. Although White argues that international collaborations may be a vehicle for infrastructural development [33], when research is funded externally, researchers may become dependent on external sources, thereby potentially deflecting the priorities from the local needs [36], [37].

Overall, the lack of funding from African governments may reflect lack of economic ability, political will or capacity to conduct intervention research [4]. The governments of low- and middle-income countries, at the Mexico Health Summit in 2005 and at the World Health Assembly in 2006, committed to spending 2% of their health budget on health research. These funds are not yet forthcoming and they need to be delivered and increased to meet the research needs of the continent [38]. In order to respond to the health priorities of the continent, African governments need to prioritize research for informing health policy decisions.

### Interventions investigated

As found in our previous study [4], there were more HIV prevention trials than treatment trials. The prevention trials were mostly dominated by interventions for the prevention of mother-to-child transmission of HIV. Few prevention trials were aimed at preventing HIV infection in sexually active adults. Many were aimed at preventing opportunistic and other infections in HIV- infected people. In treatment trials, treatment for opportunistic and other infections has led treatment research in Africa compared to pharmaceutical interventions intended for treatment of the disease itself. Antiretroviral treatment (ART) has only become available recently in many African countries and remains absent in others. The difficulty in obtaining and delivering antiretrovirals in many African settings possibly explains how few trials evaluate ART.

### Methodological quality of trials

In our study, adequate generation of the allocation sequence, allocation concealment and blinding were not well reported. This mirrors our previous results, indicating that these aspects of methodological quality has not improved during our recent study period [4]. As inadequate allocation concealment and inadequate blinding are associated with larger treatment effects [39], it is important that trials be conducted in such a way that the risk of bias is reduced and that associated methods are adequately reported. In a study of general paediatric trials published between 1948–2006, there was an increase in the methodological quality of trials over time, although most still did not report on blinding and allocation concealment even in a recent period of 2002–2006 [40].In comparing African and North American trials, Siegfried *et al*. [34] reported that African trials were more likely to report adequate generation of the allocation sequence and the allocation concealment than North American trials. This is contrary to earlier beliefs that trials of high methodological quality are not possible in settings of developing countries [41], [42]. The authors speculate that this may be due to the international collaborations driving African trials, ensuring they fulfill internationals standards [34].

Other aspects of trial quality were better reported in our study. The inclusion of a CONSORT-recommended flow diagram has been found to be associated with improved quality of trial reporting [43]. In our study 85% of reports included a flow diagram with clear loss to follow-up of participants. Another measure of trial quality is the calculation of the sample size and power of the study. Calculating the sample size prior to conducting a trial enables the researchers to enroll enough participants to answer the questions of concern, without subjecting more than the required participants to interventions that may not work, or be harmful [44]. Almost 80% of the reports of African trials in our analysis conform to this convention, showing a greater improvement compared to the earlier observations of 60% [34]. In 82% of the trials in our study, the primary outcome was clearly reported as such. Previous research has shown that in many research reports, the primary outcome is omitted, or a new outcome is introduced at the end of the study, when influenced by statistical significance already detected [45], [46]. Although we did not attempt to review protocols of the included RCTs to verify if the primary outcome reported was the intended one at the beginning of the study or not, we are nevertheless encouraged that this is increasingly well reported. As prospective clinical trial registration becomes mandatory [47], comparative analysis of protocols with final reports will strengthen studies such as ours.

### Strengths, limitations and further research

This study reviewed trials published in HIV/AIDS trials research between 2004 and 2008. The strengths of our study include the use of our fully functional Specialized Register of HIV/AIDS clinical trials which is updated quarterly from searching three major electronic databases. Our search was not limited by publication language. We used standardized methods for inclusion criteria and data extraction, and the processes were independently duplicated by two experienced reviewers. We also conducted quality control on a random sample of our trials.

This study is based on RCTs published in peer-reviewed journals and we did not search for unpublished trials from prospective clinical trials registries and conference proceedings. It is therefore possible that some trials could have been missed due to publication bias. Our register will in the near future be advancing towards including unpublished trials from conference proceedings.

The short duration of our study period did not allow us to compare trends in the HIV/AIDS- related trials for each year. Our study reviewed African RCTs, and we did not attempt to compare them with trials from other locations. This new data highlights current achievements in research and informs African stakeholders of gaps we identified. Further studies can build on this research to observe changes in methodological quality, advances in interventions over time and comparisons of these parameters with other settings.

### Conclusion

This study shows that the scope of HIV/AIDS research in Africa has not changed from our previous study including trials up to 2003. It also shows that the reporting of trial conduct has improved in some aspects. It highlights the need for African governmental and non-governmental agencies to be actively involved in funding research and for African researchers to be actively involved in leading trials.

## Supporting Information

Appendix S1 The Cochrane highly sensitive search strategy.
(DOC)

Appendix S2 The comprehensive HIV/AIDS search string.
(DOC)

Appendix S3 Sources of included trials.
(DOC)

## References

[1] World Health Organization (2010). http://www.who.int/gho/mdg/diseases/hiv/en/index.html.

[2] Chinnock P, Siegfried N, Clarke M (2005). Is evidence-based medicine relevant to the developing world?. PLoS Med.

[3] Anonymous (1998). Quality of life is rarely and poorly measured in randomised controlled trials..

[4] Siegfried N, Clarke M, Volmink J (2005). Randomised controlled trials in Africa of HIV and AIDS: descriptive study and spatial distribution.. BMJ.

[5] Glanville JM, Lefebvre C, Miles JN, Camosso-Stefinovic J (2006). How to identify randomized controlled trials in MEDLINE: ten years on.. Journal of the Medical Library Association.

[6] Namulemia E, Sparling J, Foster HD (2008). When the nutritional supplements stop: Evidence from a double-blinded, HIV clinical trial at Mengo Hospital, Kampala, Uganda.. Journal of orthomolecular medicine.

[7] Durosinmi MA, Armistead H, Akinola NO, Onayemi O, Adediran IA (2008). Selenium and aspirin in people living with HIV and AIDS in Nigeria.. Niger Postgrad Med J.

[8] Namulemia E, Sparling J, Foster HD (2007). Nutritional supplements can delay the progression of AIDS in HIV-infected patients: results from a double-blinded, clinical trial at Mengo Hospital, Kampala, Uganda.. Journal of orthomolecular medicine.

[9] Olweny CL, Borok M, Gudza I, Clinch J, Cheang M (2005). Treatment of AIDS-associated Kaposi's sarcoma in Zimbabwe: results of a randomized quality of life focused clinical trial.. Int J Cancer.

[10] Bihl F, Mosam A, Henry LN, Chisholm JV, 3rd, Dollard S (2007). Kaposi's sarcoma-associated herpesvirus-specific immune reconstitution and antiviral effect of combined HAART/chemotherapy in HIV clade C-infected individuals with Kaposi's sarcoma.. AIDS.

[11] Ross DA, Changalucha J, Obasi AI, Todd J, Plummer ML (2007). Biological and behavioural impact of an adolescent sexual health intervention in Tanzania: a community-randomized trial.. AIDS.

[12] Watson-Jones D, Weiss HA, Rusizoka M, Changalucha J, Baisley K (2008). Effect of herpes simplex suppression on incidence of HIV among women in Tanzania.. N Engl J Med.

[13] Gregson S, Adamson S, Papaya S, Mundondo J, Nyamukapa CA (2007). Impact and process evaluation of integrated community and clinic-based HIV-1 control: a cluster-randomised trial in eastern Zimbabwe.. PLoS Med.

[14] Auvert B, Taljaard D, Lagarde E, Sobngwi-Tambekou J, Sitta R (2005). Randomized, controlled intervention trial of male circumcision for reduction of HIV infection risk: the ANRS 1265 Trial.. PLoS Med.

[15] Bailey RC, Moses S, Parker CB, Agot K, Maclean I (2007). Male circumcision for HIV prevention in young men in Kisumu, Kenya: a randomised controlled trial.. Lancet.

[16] Chintu C, Bhat GJ, Walker AS, Mulenga V, Sinyinza F (2004). Co-trimoxazole as prophylaxis against opportunistic infections in HIV-infected Zambian children (CHAP): a double-blind randomised placebo-controlled trial.. Lancet.

[17] Zar HJ, Cotton MF, Strauss S, Karpakis J, Hussey G (2007). Effect of isoniazid prophylaxis on mortality and incidence of tuberculosis in children with HIV: randomised controlled trial.. BMJ.

[18] Kumwenda NI, Hoover DR, Mofenson LM, Thigpen MC, Kafulafula G (2008). Extended antiretroviral prophylaxis to reduce breast-milk HIV-1 transmission.. N Engl J Med.

[19] Goldenberg RL, Mwatha A, Read JS, Adeniyi-Jones S, Sinkala M (2006). The HPTN 024 Study: the efficacy of antibiotics to prevent chorioamnionitis and preterm birth.. Am J Obstet Gynecol.

[20] DART (2008). Fixed duration interruptions are inferior to continuous treatment in African adults starting therapy with CD4 cell counts <200 cells/microl.. AIDS.

[21] Thistle P, Spitzer RF, Glazier RH, Pilon R, Arbess G (2007). A randomized, double-blind, placebo-controlled trial of combined nevirapine and zidovudine compared with nevirapine alone in the prevention of perinatal transmission of HIV in Zimbabwe.. Clin Infect Dis.

[22] Schulz KF, Altman DG, Moher D (2010). CONSORT 2010 statement: updated guidelines for reporting parallel group randomised trials.. PLoS Med.

[23] Halpern V, Ogunsola F, Obunge O, Wang CH, Onyejepu N (2008). Effectiveness of cellulose sulfate vaginal gel for the prevention of HIV infection: results of a Phase III trial in Nigeria.. PLoS One.

[24] Chung MH, Kiarie JN, Richardson BA, Lehman DA, Overbaugh J (2008). Highly active antiretroviral therapy versus zidovudine/nevirapine effects on early breast milk HIV type-1 RNA: a phase II randomized clinical trial.. Antivir Ther.

[25] Peterson L, Taylor D, Roddy R, Belai G, Phillips P (2007). Tenofovir disoproxil fumarate for prevention of HIV infection in women: a phase 2, double-blind, randomized, placebo-controlled trial.. PLoS Clin Trials.

[26] Feldblum PJ, Adeiga A, Bakare R, Wevill S, Lendvay A (2008). SAVVY vaginal gel (C31G) for prevention of HIV infection: a randomized controlled trial in Nigeria.. PLoS One.

[27] Opara DC, Umoh BI, John M (2007). Effects of Nutritional Counseling and Micronutrient Supplementation on Some Biochemical Parameters of Persons Living with HIV and AIDS in Uyo, Nigeria.. Pakistan Journal of Nutrition.

[28] Urban MF, Bolton KD, Mokhachane M, Mphahlele RM, Bomela HN (2008). Growth of infants born to HIV-infected women when fed a biologically acidified starter formula with and without probiotics.. South African Journal of Clinical Nutrition.

[29] Isaakidis P, Swingler GH, Pienaar E, Volmink J, Ioannidis JP (2002). Relation between burden of disease and randomised evidence in sub-Saharan Africa: survey of research.. BMJ.

[30] Mystakidou K, Panagiotou I, Katsaragakis S, Tsilika E, Parpa E (2009). Ethical and practical challenges in implementing informed consent in HIV/AIDS clinical trials in developing or resource-limited countries.. SAHARA J.

[31] Ramjee G, Morar NS, Alary M, Mukenge-Tshibaka L, Vuylsteke B (2000). Challenges in the conduct of vaginal microbicide effectiveness trials in the developing world.. AIDS.

[32] Yusuf S (2002). Clinical research and trials in developing countries.. Stat Med.

[33] White MT (2007). A right to benefit from international research: a new approach to capacity building in less-developed countries.. Account Res.

[34] Siegfried N, Clarke M, Volmink J, Van der Merwe L (2008). African HIV/AIDS trials are more likely to report adequate allocation concealment and random generation than North American trials.. PLoS One.

[35] Kang DH, Davis L, Habermann B, Rice M, Broome M (2005). Hiring the right people and management of research staff.. West J Nurs Res.

[36] Benatar SR, Vaughan CL (2008). Global and local forces shaping the research agenda and the governance of research ethics.. South African Journal of Science.

[37] Siegfried N, Volmink J, Dhansay A (2010). Does South Africa need a national clinical trials support unit?. S Afr Med J.

[38] Mbewu A (2006). How academic medicine should be positioned within medicine in Africa?.

[39] Juni P, Witschi A, Bloch R, Egger M (1999). The hazards of scoring the quality of clinical trials for meta-analysis.. JAMA.

[40] Thomson D, Hartling L, Cohen E, Vandermeer B, Tjosvold L, Klassen TP (2010). Controlled trials in children: quantity, methodological quality and descriptive characteristics of pediatric controlled trials published 1948–2006.. PLoS One.

[41] Anonymous (2000). Enabling research in developing countries.. Lancet.

[42] Edejer TT (1999). North-South research partnerships: the ethics of carrying out research in developing countries.. BMJ.

[43] Egger M, Juni P, Bartlett C, for the CONSORT group (2001). Value of flow diagrams in reports of randomized controlled trials.. JAMA.

[44] Scales DC, Rubenfeld GD (2005). Estimating sample size in critical care clinical trials.. J Crit Care.

[45] Boutron I, Dutton S, Ravaud P, Altman DG (2010). Reporting and interpretation of randomized controlled trials with statistically nonsignificant results for primary outcomes.. JAMA.

[46] Chan AW, Hrobjartsson A, Haahr MT, Gotzsche PC, Altman DG (2004). Empirical evidence for selective reporting of outcomes in randomized trials: comparison of protocols to published articles.. JAMA.

[47] Abrams A, Siegfried N (2010). A Pan-African Clinical Trials Registry for the specific needs of trialists in the continent.. S Afr Med J.

