# Supplementary material for: Randomized Controlled Trials of HIV/AIDS Prevention and Treatment in Africa: Results from the Cochrane HIV/AIDS Specialized Register
Source: PLoS One. 2011 Dec 15;6(12):e28759. doi: 10.1371/journal.pone.0028759 (PMC3240627; doi:10.1371/journal.pone.0028759)
Supplement: Appendix S1 — The Cochrane highly sensitive search strategy. (DOC) [file pone.0028759.s001.doc]

**Appendix A: The Cochrane Highly Sensitive Search Strategy for identifying randomized trials in MEDLINE: sensitivity-maximizing version (2008 revision); PubMed format**

| #1  #2  #3  #4  #5  #6  #7  #8  #9  #10  #11 | randomized controlled trial [pt]  controlled clinical trial [pt]  randomized [tiab]  placebo [tiab]  drug therapy [sh]  randomly [tiab]  trial [tiab]  groups [tiab]  #1 OR #2 OR #3 OR #4 OR #5 OR #6 OR #7 OR #8  animals [mh] NOT humans [mh]  #9 NOT #10 |
| --- | --- |

*PubMed search syntax*

[pt] denotes a Publication Type term;

[tiab] denotes a word in the title or abstract;

[sh] denotes a subheading;

[mh] denotes a Medical Subject Heading (MeSH) term (‘exploded’);

[mesh: noexp] denotes a Medical Subject Heading (MeSH) term (not ‘exploded’);

[ti] denotes a word in the title.
