# Supplementary material for: Randomized Controlled Trials of HIV/AIDS Prevention and Treatment in Africa: Results from the Cochrane HIV/AIDS Specialized Register
Source: PLoS One. 2011 Dec 15;6(12):e28759. doi: 10.1371/journal.pone.0028759 (PMC3240627; doi:10.1371/journal.pone.0028759)
Supplement: Appendix S2 — The comprehensive HIV/AIDS search string. (DOC) [file pone.0028759.s002.doc]

**Appendix B: The HIV/AIDS Search String**

#1:

Search “HIV Infections”[MeSH] OR “HIV”[MeSH] OR hiv [tw] OR hiv-1*[tw] OR hiv-2*[tw] OR hiv1[tw] OR hiv2[tw] OR hiv infect*[tw] OR human immunodeficiency virus[tw] OR human immunedeficiency virus[tw] OR human immuno-deficiency virus[tw] OR human immune-deficiency virus[tw] OR ((human immun*) AND (deficiency virus[tw])) OR acquired immunodeficiency syndrome[tw] OR acquired immunedeficiency syndrome[tw] OR acquired immuno-deficiency syndrome[tw] OR acquired immune-deficiency syndrome[tw] OR ((acquired immun*) AND (deficiency syndrome[tw])) OR “Lymphoma, AIDS-Related”[MeSH] OR “Sexually Transmitted Diseases, Viral”[MeSH]

#2

Search Search “Antiretroviral Therapy, Highly Active”[MeSH] OR “Anti-Retroviral Agents”[MeSH] OR “Antiviral Agents”[MeSH:NoExp] OR ((anti) AND (hiv[tw])) OR antiretroviral*[tw] OR ((anti) AND (retroviral*[tw])) OR HAART[tw] OR ((anti) AND (acquired immunodeficiency[tw])) OR ((anti) AND (acquired immunedeficiency[tw])) OR ((anti) AND (acquired immuno-deficiency[tw])) OR ((anti) AND (acquired immune-deficiency[tw])) OR ((anti) AND (acquired immun*) AND (deficiency[tw])) OR “AIDS Vaccines”[MeSH] OR ((aids) AND (vaccin*[tw]))
