# Supplementary material for: Randomized Controlled Trials of HIV/AIDS Prevention and Treatment in Africa: Results from the Cochrane HIV/AIDS Specialized Register
Source: PLoS One. 2011 Dec 15;6(12):e28759. doi: 10.1371/journal.pone.0028759 (PMC3240627; doi:10.1371/journal.pone.0028759)
Supplement: Appendix S3 — Sources of included trials. (DOC) [file pone.0028759.s003.doc]

| **TRIAL ID** | **JOURNAL** | **YEAR** | **TITLE** |
| --- | --- | --- | --- |
| **Auvert 2005** | PLoS Medicine | 2005 | Randomized, controlled intervention trial of male circumcision for reduction of HIV infection risk: the ANRS 1256 trial |
| **Bailey 2007** | Lancet | 2007 | Male circumcision for HIV prevention in young menin Kisumi, Kenya: a randomised controlled trial |
| **Bicanic 2008** | Clinical Infectious Diseases | 2008 | High dose ampotericin B with flucytosine for the treatment of cryptococcal Meningitis in HIV-infected patients: a randomized trial |
| **Bihl 2007** | AIDS | 2007 | Kaposi’s sarcoma-associated herpes virus-specific immune reconstitution and antiviral effect of combined HAART/chemotherapy in HIV clade C-infected individuals with Kaposi’s sarcoma |
| **Bobat 2005** | Lancet | 2005 | Safety and efficacy of Zinc supplementation for children with HIV-1 infection in South Africa: a randomised double-blind placebo-controlled trial |
| **Boeree 2005** | Tropical Medicine and International Health | 2005 | Efficacy and safety of two dosages of cotrimoxazole as preventive treatment for HIV-infected Malawian adults with new smear-positive tuberculosis |
| **Celum 2008** | Lancet | 2008 | Effects of aciclovir on HIV-1 acquisition in herpes simplex virus 2 seropositive women and men who have sex with men: a randomised, double-blind, placebo controlled trial |
| **Chi 2007b** | Lancet | 2007 | Single dose tenofovir and emtricitabine for reduction of viral resistance to non-nucleoside reverse transcriptase inhibitor drugs in women given intrapartum nevirapine for perinatal HIV prevention: an open-label randomized trial |
| **Chintu 2004** | Lancet | 2004 | Co-trimoxazole as prophylaxis against oportunistic infections in HIV-infected Zambian children (CHAP): a double-blind randomized placebo-controlled trial |
| **Chung 2005** | AIDS | 2008 | Breast milk HIV-1 suppression and decreased transmission: a randomized trial comparing HIVNet 012 nevirapine versus short-course zidovudine |
| **TRIAL ID** | **JOURNAL** | **YEAR** | **TITLE** |
| **Chung 2008** | Antiviral Therapy | 2008 | Highly active antiretroviral therapy versus zidovudine/nevirapine effects on early breastmilk HIV type-1 RNA: a phase II randomized clinical trial |
| **Cornman 2008** | Journal of Acquired Immune Deficiency Syndromes | 2008 | Clinic based intervention reduces unprotected sexual behavior among HIV-infected patients in KwaZulu-Natal, South Africa: results of a pilot study |
| **Cowan 2008a** | Sexually Transmitted Diseases | 2008 | A randomised placebo-controlled trial to explore the effect of suppressive therapy with acyclovir on genital shedding of HIV-1 and herpes simplex virus type 2 among Zimbabwean sex workers |
| **DART 2008** | AIDS | 2008 | Fixed duration interruptions are inferior to continuous treatment in African adults starting therapy with CD4 cell counts < 200 cells/ml |
| **Fawzi 2005** | American Journal of Clinical Nutrition | 2005 | Trial of Zinc supplements in relation to pregnancy outcomes, hematologic indicators and T cell counts among HIV-1-infected women in Tanzania |
| **Feldblum 2008** | PLoS ONE | 2008 | SAVVY Vaginal Gel (C31G) for prevention of HIV infection: a randomized controlled trial in Nageria |
| **Friis 2004** | American Journal of Clinical Nutrition | 2004 | Effect of multimicronutrient supplementation on gestational length and birth size: a randomized, placebo-controlled, double-blind effectiveness trial in Zimbabwe |
| **Goldenberg 2006** | American Journal of Obstetrics and Gynecology | 2006 | The HTPN 024 study: the efficacy of antibiotics to prevent chorioamniotis and pre-term birth |
| **Gray 2005** | AIDS | 2005 | A randomized trial of two postexposure prophylaxis regimens to reduce mother-to-child HIV-a transmission in infants of untreated mothers |
| **Gray 2007** | Lancet | 2007 | Male circumcision for HIV prevention in men in Rakai, Uganda: a randomised trial |

| **TRIAL ID** | **JOURNAL** | **YEAR** | **TITLE** |
| --- | --- | --- | --- |
| **Gregson 2007b** | PLoS Medicine | 2007 | Impact and process evaluation of integrated community and clinic-based hiv-1 control: a cluster-randomised trial in Eastern Zimbabwe |
| **Halpern 2008** | PLoS ONE | 2008 | Effectiveness of cellulose sulfate vaginal gel for the prevention of HIV infection: results of a phase 3 trial in Nigeria |
| **Hamer 2007** | Journal of Infectious Diseases | 2007 | Two-dose versus monthly intermittent preventive treatment of malaria with sulfadoxine-pyrimethamine in HIV seropositive pregnant Zambian women |
| **Jewkes 2008** | BMJ | 2008 | Impact of Stepping Stones on incidence of HIV and HSV-2 and sexual behaviour in rural South Africa: cluster randomized controlled trial |
| **Kallestrup 2005** | Journal of Infectious Diseases | 2005 | Schistosomiasis and HIV-1 Infection in Rural Zimbabwe: Effect of Treatment of Schistosomiasis on CD4 Cell Count and Plasma HIV-1 RNA Load |
| **Kelly 2008** | American Journal of Clinical Nutrition | 2008 | Micronutrient supplementation has limited effects on intestinal infectious disease and mortality in a Zambian population of mixed HIV status: a cluster randomized trial |
| **Kuhn 2005** | AIDS | 2005 | Prolonged breast-feeding and mortality up to two years post-post partum among HIV positive women in Zambia |
| **Kuhn 2008** | NEJM | 2008 | Effects of early, abrupt weaning on HIV-free survival of children in Zambia |
| **Kumwenda 2008** | NEJM | 2008 | Extended antiretroviral prophylaxis to reduce breast-milk HIV-1 transmission |
| **Kupka 2008** | American Journal of Clinical Nutrition | 2008 | Randomized, double-blind, placebo-controlled trial of selenium supplements among HIV-infected pregnant women in Tanzania: effects on maternal and child outcomes |
| **Lightfoot 2007** | Prevention Science | 2007 | Efficacy of a culturally adapted intervention for youth living with HIV in Uganda |

| **TRIAL ID** | **JOURNAL** | **YEAR** | **TITLE** |
| --- | --- | --- | --- |
| **Luabeya 2007** | PLoS ONE | 2007 | Zinc or multiple micronutrient supplementation to reduce diarrhea and respiratory desease in South African children: a randomized controlled trial |
| **Lule 2005** | American Journal of Tropical Medicine and Hygiene | 2005 | Effect of home-based water chlorination and safe storage on diarrhea among persons with human immunodefficiency virus in Uganda |
| **Mansoor 2007** | Health Education Research | 2007 | Written medicines information for South African HIV/AIDS patients: does it enhance understanding of co-trimoxazole therapy? |
| **Mayanja-Kizza 2005** | Journal of Infectious Diseases | 2005 | Immunoadjuvant prednisolone therapy for HIV-associated tuberculosis: a phase 2 clinical trial in Uganda |
| **Mohammed 2007** | International Journal of Tuberculosis and Lung Diseases | 2007 | Randomized controlled trial of isoniazid preventive therapy in South African adults with advanced HIV disease |
| **Mutimura 2008** | Quality of Life Research | 2008 | The effects of exercise training on quality of life in HAART-treated HIV-positive Rwandan subjects with body fat redistribution |
| **Nagot 2007** | NEJM | 2007 | Reduction of HIV-1 RNA levels with therapy to suppress Herpex Simplex Virus |
| **Nduba 2008** | Thorax | 2008 | Placebo found equivalent to amoxicillin for treatment of acute bronchitis in Nairobi, Kenya: a triple blind randomized, equivalence trial |
| **Nielsen 2007** | American Journal of Tropical Medicine and Hygiene | 2007 | Effect of Diethylcarbamazine on HIV Load, CD4% and CD4/CD8 Ratio in HIV -infected adult Tanzanians with or without Lymphatic Filariasis: Randomized double-blind and placebo-controlled cross-over trial |
| **Nunn 2008** | BMJ | 2008 | Role of co-trimoxazole prophylaxis in reducing mortality in HIV infected adults being treated for tuberculosis: randomized clinical trial |
| **Olweny 2005** | International Journal of Cancer | 2005 | Treatment of AIDS-associated Kaposi's sarcoma in Zimbabwe |
| **TRIAL ID** | **JOURNAL** | **YEAR** | **TITLE** |
| **Opara 2007** | Pakistan Journal of Nutrition | 2007 | Effects of nutritional counseling and micronutrient supplementation on some biochemical parameters of persons living with HIV and AIDS in Uyo, Nigeria |
| **Padian 2007** | Lancet | 2007 | Diaphram and lubricant gel for prevention of HIV acquisition in southern African women: a randomized controlled trial |
| **Pearson 2007** | Journal of Acquired Immune Deficiency Syndromes | 2007 | Randomized control trial of peer-delivered, modified directly obsterved therapy for HAART in Mozambique |
| **Peterson 2007a** | PLoS Clinical Trials | 2007 | Tenofovir disoproxil fumarate for prevention of HIV infection in women: a phase 2, double-blind, randomized, placebo-controlled trial |
| **Pope 2008** | Journal of Acquired Immune Deficiency Syndromes | 2008 | A Cluster-Randomized Trial of Provider-Initiated (Opt-Out) HIV Counseling and Testing of Tuberculosis Patients in South Africa |
| **Prendergast 2008** | AIDS | 2008 | Early virological suppression with three-class antiretroviral therapy in HIV-infected African infants |
| **Rollins 2007** | Acta Paediatrica | 2007 | The effect of nutritional support on weight gain of HIV-infected children with prolonged diarrhoea |
| **Ross 2007** | AIDS | 2007 | Biological and behavioural impact on adolescent sexual health intervention in Tanzania: a community randomized trial |
| **Sarna 2008** | Epidemiology and Social Science | 2008 | Short- and long-term efficacy of modified directly obsterved antiretroviral treatment in Mombasa, Kenya: a randomized trial |
| **Scarborough 2007** | NEJM | 2007 | Corticosteroids for bacterial meningitis in adults in Sub-Saharan Africa |
| **Sebitloane 2008** | American Journal of Obstetrics and Gynecology | 2008 | Prophylactic antibiotics for the prevention of postpartum infectious morbidity in women infected with human immunedefficiency virus: a randomized controlled trial |
| **Semba 2007a** | International Journal of Tuberculosis and Lung Dis | 2007 | Micronutrient supplements and mortality of HIV-infected adults with pulmonary TB: a controlled clinical trial |

| **TRIAL ID** | **JOURNAL** | **YEAR** | **TITLE** |
| --- | --- | --- | --- |
| **Skoler-Karpoff 2008** | Lancet | 2008 | Efficacy of carraguard for prevention of HIV infection in women in South Africa: a randomised, double-blind, placebo-controlled trial |
| **Stephenson 2008** | AIDS Care | 2008 | The influence of motivational messages on future planning behaviors among HIV concordant positive and discordant couples in Lusaka, Zambia |
| **Stringer 2007** | American Journal of Obstetrics and Gynecology | 2007 | A randomized trial of the intrauterine contraceptive devide vs hormonal contraception in women who are infected with the human immunodefficiency virus |
| **SWEN 2008** | Lancet | 2008 | Extended-dose nevirapine to 6 weeks of age for infants to prevent HIV transmission via breastfeeding in Ethiopia, India, and Uganda: an analysis of three randomized controlled trials |
| **Taha 2004** | JAMA | 2004 | Nevirapine and zidovudine at birth to reduce perinatal transmission of HIV in an African setting |
| **Taha 2007b** | PLoS Clinical Trials | 2007 | Intermittent intravaginal antibiotic treatment of bacterial vaginosis in HIV-unifected and infected women: a randomized clinical trial |
| **Thior 2006** | JAMA | 2006 | Breastfeeding plus infant zidovudine prophylaxis for 6 months vs formula feeding plus infant zidovudine for 1 month to reduce mother-to-child HIV transmission in Botswana: a randomized trial: The Mashi study |
| **Thistle 2007** | Clinical Infectious Diseases | 2007 | A randomized, Double-Blind, Placebo-Controlled Trial of combined Nevirapine and Zidovudine compared with Nevirapine alone in the prevention of perinanal transmission of Hiv in Zimbabwe |
| **Urban 2008** | South African Journal of Clinical Nutrition | 2008 | Growth of infants born to HIV-infected womenwhen fed a biologically acidified starter formula with and without probiotics |
| **Van Damme 2008** | NEJM | 2008 | Lack of effectiveness of the cellulose sulfate gel for the prevention of vaginal Hiv transmission |
| **TRIAL ID** | **JOURNAL** | **YEAR** | **TITLE** |
| **Villamor 2008** | Journal of Infectious Diseases | 2008 | A trial of the effects of micronutrient supplementation on treatment outcome, T cell counts, morbidity and mortality in adults with pulmonary tuberculosis |
| **Watson-Jones 2008** | NEJM | 2008 | Effect of herpes simplex suppression on incidence of HIV among women in Tanzania |
| **Zar 2007** | BMJ | 2007 | Effect of isoniazid prophylaxis on mortality and incidence of tuberculosis in children with HIV: randomised controlled trial |
| **Zulu 2005** | Alimentary pharmacology and therapeutics | 2005 | Nitazoxanide for persistent diarrhoea in Zambian acquired immune defficiency syndrome patients: a randomized-controlled trial |
